# Distribution of Awards

**Published:** 1916-08-19

**Authors:** 


### METROPOLITAN HOSPITAL SUNDAY FUND.

### Distribution of Awards.

A meeting of the Council of the Fund was held at the Mansion House on Tuesday, the 15th inst., under the presidency of the Right Hon. the Lord Mayor (Sir Charles C. Wakefield).

Immediately on taking the chair the Lord Mayor expressed the great pleasure it gave him to see again Viscount Knutsford after his severe accident.

The Secretary intimated that letters of regret for absence had been received from the Bishop of London, the Bishop of Islington, the Archbishop of Westminster, the Archdeacons of London, Westminster, and Hampstead, Canon Allen Bell, Dr. Fleming, the Chief Rabbi, Sir Richard Martin, Sir Alfred Pearce Gould, Mr. J. Henry Buxton, etc.

The Chairman, in a few introductory remarks, said he had come up from the country specially to attend the meeting, as he had always felt that the presidency of the Hospital Sunday Fund was one of the most important of the positions attaching to the office of Lord Mayor. He was glad to know that although the receipts of the Fund for this year totalled some £2,800 less than last year, yet the amount was, with that exception, the largest amount raised during the last seven years, a very satisfactory result when one considered the times in which we were now living, and the enormous sums being devoted to charity in various forms. He expected to hear that the hospitals had been very hard hit over the war, not only by the calling up of their medical and civil staffs, but by the great increase in the prices of drugs and the competition of war charities. Possibly there might be some alleviating circumstances. He read that at one important Metropolitan hospital there had been a large falling off in the number of out-patients, doubtless owing to the increased wages earned by the working classes, the disappearance of unemployment, and the tendency to make light of ailments which in normal times might have been regarded as more important. In conclusion, he expressed his warm thanks to the clergy and ministers of churches who had instituted special collections for the Fund, and pleaded the cause of the hospitals. He attended three services on Hospital Sunday—Westminster Abbey, St. Paul's Cathedral, and Union Chapel—and he was struck by the interest taken in the Fund by the large congregations assembled at all three places of worship.

Sir William Church, Bart, K.C.B., proposed: "That the report of the Committee of Distribution for the year 1916 be, and the same is hereby, approved, and that the awards thereby recommended be paid forthwith."

### A Satisfactory Year.

Sir Ernest Tritton, Bart., seconded. He thought that the year, under the peculiar circumstances, had been a very satisfactory one indeed. The fact that the Fund was only £2,800 less than the high total of last year showed that the people of London still had a very deep and sincere regard for their hospitals, a regard which had been intensified during the past two years by the fact that so many gallant wounded sailors and soldiers had been treated and carefully tended within hospital wards. Those patients had shown a bravery and a heroism which was unequalled in the history of the world. £66,430 10s. had been distributed among 248 institutions.

Viscount Knutsford proposed: "That the best thanks of the Council be, and are hereby, accorded to the Committee of Distribution for the care they have bestowed upon the preparation of the awards," and he would have liked the name of Sir Ernest Tritton to be specially included in the resolution, because of his work as Vice-Chairman of the Fund, which entailed a great deal of time and energy. It was something to be able to say that during all the years that Sir William Church had presided over the sittings of the Distribution Committee, the awards had been received without any comment at all, but with a feeling of real gratitude. It was not a very easy matter to please 2,200 ministers of every form of religion, yet that was what the Distribution Committee had succeeded in doing. He wished also to compliment the Distribution Committee on not making what was known as the cost per bed the basis of the awards. It was a great mistake to suppose that a hospital was badly managed because its cost per bed was high, or that it was necessarily well managed because the cost per bed was low. What was necessary to know, and that the Distribution Committee had taken the trouble to find out, was what the hospital was doing with its beds. If the cost per bed at a particular hospital went up, the Committee of the Fund asked why. He had given evidence on such occasions, and each time he left the Court without a stain on his character. (Laughter.)

Sir Joseph Savory seconded the motion with great pleasure, and hoped the vote would not be regarded as a formal one, for there was a feeling of heartfelt gratitude to Sir William Church, Sir Ernest Tritton, and the Committee generally for all they had done for the Fund.

Sir Marcus Samuel proposed that the thanks of the Council be tendered to the editors of newspapers who had pleaded the cause of the hospitals and of this Fund in particular. Mr. J. R. Cooper (Ex-Sheriff) seconded this motion, and it was carried.

Lord Meath, in proposing that the cordial thanks of the Council be accorded to the Right Hon. the Lord Mayor, said he feared people were liable to forget what an enormous amount of labour was entailed upon the Lord Mayor in connection with his patronage of the Fund, but it would be thrown into greater relief if a successor to that high office were not to take the same active part in it which so many of his predecessors had done.

Dr. Parker Young seconded, and it was carried.

The Lord Mayor, in acknowledging the vote, declared he found his activities in connection with the Fund truly a labour of love.

The meeting next proceeded to the consideration of the report of the Distribution Committee, and the awards (see pp. 478, 479), which were approved.

### A Fixed Date for Hospital Sunday?

The Rev. W. Hardy Harwood moved: "That the General Purposes Committee be asked to consider the practicability of establishing a fixed date for Hospital Sunday (preferably the second Sunday in June), and to report to the Council before the annual meeting of constituents in December." He said the question was whether any change was desirable, in the interests of the Fund, or whether it was better for the arrangement which had been operative for forty years to be perpetuated. At present the collection for the Fund was taken on the First Sunday after Trinity, which, he believed, originated in some special service at St. Paul's Cathedral. The date was, however, a movable one. It might be urged that full notice of the date was given, but all clergy were not equally businesslike; and he firmly believed it would be better for the Fund if the collection were to be taken on a definite fixed day, especially as it was usual for Churches to make their arrangements a year in advance. There would also be some sentiment in having a certain day every year devoted to the sacred cause of the hospitals. It was not desirable to change from the month of June. Possibly the third Sunday in the month would be better than the second, but that was a matter for consideration.

Sir Marcus Samuel seconded the proposition, and expressed himself as in accord with the remarks of the proposer. At the present day there were many conflicting collections, and he remembered one which was taken up on a date so close before Hospital Sunday that it seemed inevitable that part of the money given to the rival charity would otherwise have gone to swell this Fund.

Viscount Knutsford moved the addition of the words, after "June," "or some earlier date in the year." His object was to invite the General Purposes Committee to consider whether it was wise to continue to have Hospital Sunday in the summer-time at all. He thought it very doubtful whether it was in the best interests of the Fund to continue the Sunday in June. Certainly it would be a mistake to fix it as the second Sunday in June, because that might be Trinity Sunday in some years, a day on which many clergy would not like to take up a special collection. His experience in begging had taught him that people gave better in cold than in warm weather; a more natural and direct appeal could be made in cold weather. Moreover, attendance at churches was better in winter than in summer. Another point to bear in mind was that June was the month in which many charities held garden parties, and made street collections. General charity activities were not so pronounced before Easter, and he suggested that would be a good time of year at which to hold Hospital Sunday.

The proposer and seconder offered no objection to the proposal; it was therefore added to the original resolution and agreed to.

A letter was read from the Rev. F. W. Newland, of the Claremont Central Mission of the London Congregational Union, protesting against the practice of some hospitals and convalescent institutions limiting the validity of letters purchased by subscribers to a particular year, and refusing to receive a patient upon such letter if the due time had expired. He asked that the Fund would take steps to have this restriction removed.

The matter was referred to the General Purposes Committee.

The meeting concluded by the passing of resolutions of thanks to the Lord Mayor for presiding, and to the Secretaries (Messrs. James and Lander) for their admirable work on the Fund.

### REPORT OF THE COMMITTEE OF DISTRIBUTION TO THE COUNCIL.

The Committee of Distribution held their first meeting on Friday, May 26, and then and at subsequent meetings examined the reports and income and expenditure accounts of the several institutions seeking to participate, and made their awards in conformity with the Laws of the Constitution.

The Committee beg to report that the total of the Fund on August 15 will amount to £68,000.

This year 248 institutions have applied to participate in the Fund, being two more than in 1915, and the Committee now recommend the distribution of £66,430 10s. to the several hospitals, institutions, dispensaries, and nursing associations shown in the appendix hereto.

The Committee having reduced the bases of award to eight institutions, the governing bodies of the same were informed thereof in accordance with the Laws of the Constitution. Seven deputations attended the Committee, and after hearing their several explanations the bases of award were finally determined.

Seven and a-half per cent. of the total sum available for distribution is appropriated to the purchase of surgical appliances during the ensuing year, and 2½ per cent. for district nursing associations.

In compliance with an order of the Council, statistics have been prepared as usual for the use of its members, showing the number of beds in hospitals, the cost of patients at hospitals and dispensaries, the proportionate expense of management to maintenance, and other information. A copy thereof has been sent to each member of the Council.

Charles Cheers Wakefield, Lord Mayor, President and Treasurer.
C. Ernest Tritton, Vice-Chairman.
William S. Church, Chairman of Committee.
David Anderson. G. H. Makins.
W. Hardy Harwood. Adrian Pollock.
Somerleyton. Marcus Samuel.
F. A. Bevan. E. Parker Young.
Richard B. Martin, John Henry Buxton, } Hon. Secs.

The Mansion House, July 31, 1916.

### Awards Recommended by the Committee of Distribution for the Year 1916,

THIRTY-TWO GENERAL HOSPITALS.

Charing Cross Hospital, £1,365 12s. 6d.; French Hospital, £229 0s. 10d.; Great Northern Central Hospital, £1,464 6s. 8d.; Guy's Hospital, £2,100 13s. 4d.; Hampstead and N.W. London Hospital, £837 11s. 8d.; Italian Hospital, £193 11s. 8d.; Kensington General Hospital, £74 15s.; King's College Hospital, £1,765 5s.; London Hospital, £7,250 15s.; London Homœopathic Hospital, £736 19s. 2d.; Phillips' Memorial Homœopathic Hospital, £53 13s. 4d.; London Temperance Hospital, £663 3s. 4d.; Metropolitan Hospital, £1,213 5s.; Mildmay Hospital, £270 5s.; Mildmay Memorial Hospital, £69; Miller Hospital and Royal Kent Dispensary, £566 7s. 6d.; Poplar Hospital, £539 10s. 10d.; Prince of Wales' Hospital, £991 17s. 6d.; Royal Free Hospital, £1,397 5s.; St. George's Hospital, £2,375 14s. 2d.; S.s. John and Elizabeth Hospital, £149 10s.; St. John's Hospital, Lewisham, £249 3s. 4d.; St. Mary's Hospital, £1,929 2s. 6d.; St. Thomas's Hospital, £943 19s. 2d.; Seamen's Hospital Society, £1,382 17s. 6d.; The Middlesex Hospital and Convalescent Home, £3,034 1s. 8d.; University College Hospital, £1,833 5s. 10d.; Walthamstow, &c., Hospital, £152 7s. 6d.; Wandsworth, Bolingbroke Hospital, £374 14s. 2d.; West Ham Hospital, £1,039 15s. 10d.; West London Hospital, £1,358 18s. 4d.; Westminster Hospital, £1,462 8s. 4d.

SIX CHEST HOSPITALS.

City of London Hospital for Diseases of the Chest, Victoria Park, £841 8s. 4d.; Hospital for Consumption, Brompton, £2,115 0s. 10d.; Mount Vernon Consumption Hospital, £219 9s. 2d.; Royal Hospital for Diseases of the Chest, £552 19s. 2d.; Royal National Sanatorium, £14 7s. 6d.; Royal National Hospital, Ventnor, £172 10s.

TWENTY CHILDREN'S HOSPITALS.

Alexandra Hospital for Hip Disease, £216 11s. 8d.; Banstead Surgical Home, £28 15s.; Barnet Home Hospital, £33 10s. 10d.; Belgrave Hospital, £297 1s. 8d.; Cheyne Hospital for Incurable Children, £170 11s. 8d.; East London Hospital for Children, £858 13s. 4d.; Evelina Hospital for

Sick Children, £230 19s. 2d.; Home for Incurable Children, £39 5s. 10d.; Home for Sick Children, Sydenham, £120 15s.; Hospital for Sick Children, £1,388 12s. 6d.; Infants' Hospital, Westminster, £253; Kensington, for Children, and Dispensary, £73 15s. 10d.; Paddington Green Hospital for Children, £317 4s. 2d.; Queen's Hospital for Children, £1,334 19s. 2d.; Royal Waterloo Hospital, Children and Women, £604 14s. 2d.; St. Mary's Hospital, Plaistow, £309 10s. 10d.; St. Monica's Hospital, Brondesbury, £79 10s. 10d.; Victoria Hospital for Children, Chelsea, £630 11s. 8d.; Victoria Home, Margate, £48 17s. 6d.; Hospital for Hip Disease, Sevenoaks, £52 14s. 2d.

### SEVEN LYING-IN HOSPITALS.

British Home for Mothers and Babies, £46; City of London Lying-in Hospital, £122 13s. 4d.; Clapham Maternity Hospital and Dispensary, £23 19s. 2d.; East End Mothers' Home, £81 9s. 2d.; General Lying-in Hospital, Lambeth, £191 13s. 4d.; Plaistow Maternity Hospital, £36 8s. 4d.; Queen Charlotte's Lying-in Hospital, £509 16s. 8d.

### SIX HOSPITALS FOR WOMEN.

Chelsea Hospital for Women, £335 8s. 4d.; Hospital for Women, Soho Square, £431 5s.; Grosvenor Hospital for Women and Children, £143 15s.; New Hospital for Women, £424 10s. 10d.; Samaritan Free Hospital, £484 18s. 4d.; South London Hospital, £32 11s. 8d.

### TWENTY-FOUR OTHER SPECIAL HOSPITALS.

Middlesex Hospital, Cancer Wing, £237 13s. 4d.; London Fever Hospital, Islington, £239 11s. 8d.; Gordon Hospital for Fistula, £19 3s. 4d.; St. Mark's Hospital for Fistula, £287 10s.; National Hospital for the Diseases of the Heart, £124 11s. 8d.; Female Lock Hospital, Harrow Road, £324 17s. 6d.; Hospital for Epilepsy, Paralysis, etc., £239 11s. 8d.; National Hospital for the Paralysed and Epileptic, £1,012; West End Hospital for Diseases of the Nervous System, £313 7s. 6d.; Central London Ophthalmic Hospital, £191 13s. 4d.; Royal Eye Hospital, £193 11s. 8d.; Royal London Ophthalmic Hospital, £996 13s. 4d.; Royal Westminster Ophthalmic Hospital, £108 5s. 10d.; Western Ophthalmic Hospital, £98 14s. 2d.; Royal National Orthopædic Hospital, £641 2s. 6d.; Royal Sea Bathing Hospital, Margate, £95 16s. 8d.; St. John's Hospital for Diseases of the Skin, £42 2s. 6d.; St. Peter's Hospital for Stone, £135 2s. 6d.; Central London Throat and Ear Hospital, £35 9s. 2d.; Throat Hospital, Golden Square, £69 19s. 2d.; London Throat Hospital, £37 7s. 6d.; Metropolitan Ear, Nose, and Throat Hospital, £63 5s.; Royal Dental Hospital of London, £188 15s. 10d.; National Dental Hospital, £68 0s. 10d.

### THIRTY-TWO CONVALESCENT HOSPITALS.

Metropolitan Convalescent Institution, Walton, £345; Metropolitan Convalescent Institution, Broadstairs, £271 4s. 2d.; Metropolitan Convalescent Institution, Bexhill, £504 1s. 8d.; All Saints' Convalescent Hospital, Eastbourne, £383 6s. 8d.; All Saints' Convalescent Home, St. Leonards, £25 17s. 6d.; Ascot Priory Convalescent Home, £57 10s.; Brentwood Convalescent Home for Children, £15 6s. 8d.; Chelsea Hospital for Women Convalescent Home, St. Leonards, £55 11s. 8d.; Mrs. Gladstone's Convalescent Home, Mitcham, £82 8s. 4d.; Friendly Societies' Convalescent Home, Dover, £115; Hahnemann Convalescent Home, Bournemouth, £18 4s. 2d.; Hanwell Convalescent Home, £2 17s. 6d.; Hastings, Fairlight Sanatorium, £7 13s. 4d.; Herbert Convalescent Home, Bournemouth, £9 11s. 8d.; Herne Bay Baldwin-Brown Convalescent Home, £47 18s. 4d.; Homœopathic Hospital Convalescent Home, £3 16s. 8d.; Hemel Hempstead Convalescent Home, £18 4s. 2d.; Mary Yolland Home, £16 5s. 10d.; Mrs. Kitto's Convalescent Home, Reigate, £41 4s. 2d.; London Hospital Convalescent Home, Tankerton, £68 0s. 10d.; Police Seaside Home, Brighton, £54 12s. 6d.; St. Andrew's Convalescent Home, Clewer, £57 10s.; St. Andrew's Convalescent Home, Folkestone, £162 18s. 4d.; St. John's Home for Convalescent and Crippled Children, £22 0s. 10d.; St. Joseph's Convalescent Home, Bournemouth, £14 7s. 6d.; St. Leonards-on-Sea Convalescent Home for Poor Children, £115; Eversfield, St. Leonard's, £5 15s.; St. Mary's Convalescent Home, Birchington, £19 3s. 4d.; St. Mary's Convalescent Home, Broadstairs, £143 15s.; St. Mary's Convalescent Home, Shortlands, £16 5s. 10d.; St. Michael's Convalescent Home, Westgate, £20 2s. 6d.; Seaside Convalescent Hospital, Seaford, £47 18s. 4d.

### TWENTY-EIGHT COTTAGE HOSPITALS.

Acton Cottage Hospital, £101 11s. 8d.; Beckenham Cottage Hospital, £89 2s. 6d.; Blackheath and Charlton Cottage Hospital, £106 7s. 6d.; Brentwood Cottage Hospital, £40 5s.; Bromley, Kent, Cottage Hospital, £104 9s. 2d.; Bushey Heath Cottage Hospital, £40 5s.; Canning Town Cottage Hospital, £113 1s. 8d.; Chislehurst, Sidcup and Cray Cottage Hospital, £73 15s. 10d.; Coldash Cottage Hospital, £45 0s. 10d.; Ealing Cottage Hospital, £146 12s. 6d.; East Ham Cottage Hospital, £68 0s. 10d.; Eltham Cottage Hospital, £64 4s. 2d.; Enfield Cottage Hospital, £91 0s. 10d.; Epsom and Ewell Cottage Hospital, £53 13s. 4d.; Hendon Cottage Hospital, £43 2s. 6d.; Hounslow Cottage Hospital, £57 10s.; Livingstone Dartford Cottage Hospital, £122 13s. 4d.; Kingston, Victoria Hospital, £63 5s.; Molesey and Hampton Court Cottage Hospital, £14 7s. 6d.; Reigate and Redhill Hospital, £187 16s. 8d.; Sidcup Cottage Hospital, £31 12s. 6d.; Surbiton Cottage Hospital, £35 9s. 2d.; Teddington Cottage Hospital, £47 18s. 4d.; Tilbury (Passmore Edwards) Cottage Hospital, £47 18s. 4d.; Willesden Cottage Hospital, £114 0s. 10d.; Wimbledon Cottage Hospital, £86 5s.; Wimbledon Nelson Hospital, £69; Wood Green Cottage Hospital, £76 13s. 4d.

### TWELVE INSTITUTIONS.

Florence Nightingale Hospital for Invalid Gentlewomen, £125 10s. 10d.; St. Andrew's, Dollis Hill, £26 16s. 8d.; St. Saviour's Hospital and Nursing Home, £27 15s. 10d.; Invalid Asylum, Stoke Newington, £46; Firs Home, Bournemouth, £16 5s. 10d.; St. Columba's Hospital, £275 0s. 10d.; Free Home for Dying, Clapham, £9 11s. 8d.; St. Luke's House, Pembridge Square, £110 4s. 2d.; Santa Claus Home, Highgate, £72 16s. 8d.; Royal Mineral Water Hospital, Bath, £23 9s. 2d.; Winifred House, Holloway, £51 15s.; Highgate Convalescent Home, £43 2s. 6d.

### FORTY-FIVE DISPENSARIES.

Battersea Provident Dispensary, £142 15s. 10d.; Billingsgate Dispensary, £48 17s. 6d.; Brixton, etc., Dispensary, £42 3s. 4d.; Buxton Street Dispensary, £9 11s. 8d.; Camberwell Provident Dispensary, £46 19s. 2d.; Childs Hill Provident Dispensary, £4 15s. 10d.; City Dispensary, £64 4s. 2d.; Clapham General and Provident Dispensary, £20 2s. 6d.; Eastern Dispensary, £32 11s. 8d.; East Dulwich Provident Dispensary, £45 0s. 10d.; Farringdon General Dispensary, £38 6s. 8d.; Finsbury Dispensary, £54 12s. 6d.; Forest Hill Provident Dispensary, £33 10s. 10d.; Greenwich Provident Dispensary, £23 19s. 2d.; Hampstead Provident Dispensary, £37 7s. 6d.; Islington Dispensary, £41 4s. 2d.; Islington Medical Mission, £50 15s. 10d.; Kentish Town Medical Mission, £18 4s. 2d.; Kilburn, Maida Vale, and St. John's Wood Dispensary, £33 10s. 10d.; Kilburn Provident Medical Institution, £42 3s. 4d.; London Dispensary, Spitalfields, £12 9s. 2d.; London Medical Mission, Endel Street, £87 4s. 2d.; Margaret Street, for Consumption, £46 19s. 2d.; Metropolitan Dispensary, £26 16s. 8d.; Mildmay Medical Mission Dispensary, £13 8s. 4d.; Notting Hill Provident Dispensary, £19 3s. 4d.; Paddington Provident Dispensary, £26 16s. 8d.; Public Dispensary, £39 5s. 10d.; Queen Adelaide's Dispensary, £15 6s. 8d.; Royal General Dispensary, £21 1s. 8d.; Royal Pimlico Provident Dispensary, £23; Royal South London Dispensary, £35 9s. 2d.; St. George's, Hanover Square, Dispensary, £24 18s. 4d.; St. John's Wood Provident Dispensary, £38 6s. 8d.; St. Marylebone General Dispensary, £63 5s.; St. Pancras and Northern Dispensary, £39 5s. 10d.; South Lambeth, Stockwell, and North Brixton Dispensary, £23 19s. 2d.; Stamford Hill, Stoke Newington

Dispensary, £51 15s.; Wandsworth Common Provident Dispensary, £7 13s. 4d.; Westbourne Provident Dispensary, £12 9s. 2d.; Western Dispensary, £43 2s. 6d.; Western General Dispensary, £71 17s. 6d.; Westminster General Dispensary, £31 12s. 6d.; Whitechapel Provident Dispensary, £18 4s. 2d.; Woolwich Provident Dispensary, £28 15s.

THIRTY-THREE NURSING ASSOCIATIONS.

Belvedere, Abbey Wood, £6; Brixton, £30; Central St. Pancras, £30; Charlton and Blackheath, £6; Chelsea and Pimlico, £18; Hackney, £36; Hammersmith, £60; Hampstead, £18; Isleworth, £12; Kensington, £54; Kilburn, £6; Kingston, £42; Lambeth Road (Catholic), £6; Metropolitan (Bloomsbury), £48; St. Olave's (Bermondsey), £30; Paddington and Marylebone, £48; Plaistow, £62 8s.; Plaistow (Maternity), £96; Ponders End, Enfield, etc., £12; Rotherhithe, £12; Shoreditch, £60; Sick Room Helps Society, £18; Sidcup, £6; Silvertown, £18; South London (Battersea), £54; Southwark, £42; South Wimbledon, £30; Tottenham, £24; Westminster, £18; Woolwich, £42; East London, £78; North London, £54; Ranyard Nurses, £372.

